# The Lepidoptera of White Sands National Monument, Otero County, New Mexico, USA 3. A new species of *Aleptina* Dyar, 1902 (Lepidoptera, Noctuidae, Amphipyrinae, Psaphidini)

**DOI:** 10.3897/zookeys.149.1517

**Published:** 2011-11-24

**Authors:** Eric H. Metzler, Gregory S. Forbes

**Affiliations:** 1 Adjunct Curator of Lepidoptera, Michigan State University, P.O. Box 45, Alamogordo, NM 88311-0045 U.S.A.; 2 1009 Luna St, Las Cruces, New Mexico 88001 U.S.A.

**Keywords:** Tularosa Basin, biological diversity, white gypsum dunes, Noctuidae, White Sands National Monument, New Mexico, National Park, Otero County

## Abstract

In 2006 the US National Park Service initiated a long-term study of the Lepidoptera at White Sands National Monument, Otero County, New Mexico. *Aleptina arenaria* **sp. n.**, described here, was discovered in 2008, the second year of the study. The adult moths and male and female genitalia are illustrated.

## Introduction

The North American genus *Aleptina* Dyar (1902) was revised by Todd et al. (1984). In 2008, 2009, and 2010, adults of an unknown species of *Aleptina* were collected at White Sands National Monument, New Mexico. No specimens of this species were known prior to this study of insects at the Monument. The lack of specimens can probably be attributed to a dearth of collecting in the gypsum dunes ecosystem of White Sands National Monument in New Mexico, which is under the jurisdiction of the U.S. National Park Service and the U.S. Army’s White Sands Missile Range.

## Methods

More than 250 samples of moths and other night flying insects were collected in U.S.D.A. type black light traps, and at black light, as described in Covell (1984), sometimes with mercury vapor light, and sheet on more than 75 different nights. A detailed description of the study methods is in Metzler et al. (2009). Genitalia were examined following procedures outlined in Metzler and Forbes (2011). Terminology for elements of wing pattern, morphology, and genital structures follows Todd et al. (1984), Lafontaine (2004), and Mikkola et al. (2009).

All specimens were collected as part of a long-term study of Lepidoptera at White Sands National Monument, and they are deposited in the following collections:

EHM Eric H. Metzler, Alamogordo, NM, for subsequent transfer to MSU


MSU Albert J. Cook Arthropod Research Collection, Department of Entomology, Michigan State University, East Lansing, MI


UNM Museum of Southwestern Biology, University of New Mexico, Albuquerque, NM


USNM United States Museum of Natural History (Smithsonian Institution), Washington, DC pending mutual resolution and agreement with the National Park Service regarding specimen deposition.


## Results

### Aleptina arenaria

Metzler & Forbes, sp. n.

urn:lsid:zoobank.org:act:B2D5BDEB-3743-4C92-87EE-9899390FA809

http://species-id.net/wiki/Aleptina_arenaria

Figs 1, 2 11, 12 13, 14 17, 18

#### Type Material.

 Holotype: male, pinned with labels as follows: “USA: NM: Otero Co. White Sands Nat[ional] Mon[ument], interdune habitat, 106°10.84'W, 32°46.64'N 4,008', 17 May 2010 WsnmF, Eric H. Metzler, uv tr[a]p, Accss #: WHSA - 00131" “HOLOTYPE USNM *Aleptina arenaria* Metzler & Forbes” [red handwritten label] (USNM). Paratypes: 5 males and 7 females: USA: NM: Otero Co. White Sands Nat[ional] Mon[ument] (hereafter WSNM), interdune habitat, 106°11.38'W, 32°46.69'N 4,000', 15 July 2009 WSNM8, Eric H. Metzler, uv tr[a]p, Accss #: WHSA - 00131. USA: NM: Otero Co. WSNM, interdune habitat, 106°10.84'W, 32°46.64'N 4,008’, 12 Sept. 2010 WSNMF, Eric H. Metzler, uv tr[a]p, Accss #: WHSA – 00131. WSNM, interdune habitat, 106°11.38'W, 32°46.69'N 4,000', 11 June 2010 WSNM8, Eric H. Metzler, uv tr[a]p, Accss #: WHSA - 00131. WSNM, interdune habitat, 106°11.32'W, 32°45.72'N 4,000', 22 July 2008 WSNM9, Eric H. Metzler, uv tr[a]p, Accss #: WHSA - 00131. WSNM, interdune habitat, 106°11.32'W, 32°45.72'N 4,000', 17 May 2010 WSNM9, Eric H. Metzler, uv tr[a]p, Accss #: WHSA - 00131. WSNM, interdune habitat, 106°10.84'W, 32°46.64'N 4,008', 14 Sept 2009 WsnmF, Eric H. Metzler, uv tr[a]p, Accss #: WHSA - 00131. WSNM, interdune habitat, 106°10.82'W, 32°46.62'N 4,008', 15 July 2009 WSNMD, Eric H. Metzler, uv tr[a]p, Accss #: WHSA - 00131. Paratypes are deposited with UNM, MSU and EHM.

#### Etymology.

 Gypsum sand is the substrate of the white dune field at White Sands National Monument. *Arena* means sand in Latin. The Latin suffix -aria means connected with something. The specific epithet of this species, *arenaria*, a singular adjective, calls attention to the specialized sandy habitat where *Aleptina arenaria* was discovered.

#### Diagnosis.

*Aleptina arenaria* (Figs 1, 2) is a gray moth with normal noctuid transverse markings and spots. The diagnostic features are 1) male with gray hindwings, 2) antemedial and subterminal areas of the forewing are pale tan (chamois colored), 3) smoothly rounded apex of the male valve without process (Fig. 11), and 4) blunt posteriorly directed processes on female 8^th^ sternite (Figs 13, 14). In White Sands National Monument *Aleptina arenaria* flies with and might be mistaken for a washed-out specimen of *Aleptina inca* Dyar, 1913 (Figs 3, 4).The male hind wing of *Aleptina inca* is white, the male hindwing of *Aleptina arenaria* is gray. The clasper of the male genitalia of *Aleptina inca* (Fig. 7) is half the length of the valve and slender; the clasper of *Aleptina arenaria* is shorter and heavier, more like that of A*leptina clinopetes* (Dyar, 1920) (Figs 5, 6, 9). The costal margin at the distal end of the valve of *Aleptina clinopetes* (Fig. 9) has a finger-like process; the costal margin at the distal end of the valve of *Aleptina arenaria* is without a process. The posteriorly directed processes of the female 8^th^ sternite of *Aleptina inca* (Fig. 15) are thin, long, and pointed; the processes of *Aleptina arenaria* are stout and short. In *Aleptina clinopetes*, the processes are stout and longer (Fig. 16). In the key to species in Todd et al. (1984), *Aleptina arenaria* keys to *Aleptina inca* in couplet 1 because both species have the front (frons) produced into a shelf-like prominence. The differences noted above will separate *Aleptina arenaria*, *Aleptina inca*, and *Aleptina clinopetes*.

#### Description.

 Adult male (Fig. 1). Head - frons produced into a shelf, gray dorsad, pale tan (chamois colored) between the shelf and clypeus; vertex scales narrow strap-like, gray; labial palpus erect, scales spatulate, black laterally and dorsally, white ventrally, each segment pale tipped. Haustellum coiled between labial palpi. Antenna filiform, dorsally pale gray, closely scaled, ventrally naked, gray. Thorax - dorsum gray, mixture of pale and dark, scales spatulate; underside dirty white, scales erect long hair-like. Legs dark gray, closely scaled, sparse long hair-like whitish scales; tarsomeres dark gray and black, white tipped. Fore wing: Length 10 mm (no variation, n = 4). Gray with pale tan (chamois colored) in antemedial and subterminal areas. Basal line obscure, basal area pale gray; antemedial line double at costa, single at inner margin, antemedial area pale tan; postmedial line obscure, excurved beyond reniform, highlighted with dark dashes on veins, double at inner margin; subterminal line a white shade, postmedial area pale tan; terminal line black, subterminal area gray; orbicular spot a round black and white ring filled with gray; reniform spot a kidney shaped black and white ring filled with gray; costa at postmedial area pale gray; fringe gray with white bars; underside gray, darker along costal and terminal areas; fringe dark gray. Hind wing pale gray, darker in terminal areas, fringe pale gray; underside pale gray, costa darker, fringe pale gray. Abdomen - dorsum closely scaled, pale tan-gray; underside closely scaled, whitish. Genitalia (Fig. 11) - tegumen expanded laterally, bilobed, uncus nearly straight, lightly setose, terminally produced to a small bulb, three stout dorsal setae; saccus short, narrowly V-shaped; juxta with long thin spine pointed anteriorly; valve strap-like, sacculus sclerotized, well developed, costa undulating, sclerotized basal 2/3 length, cucullus membranous, corona not developed, clasper sinuous, 1/3 length of valve, stout at base, produced to a point. Aedeagus (Fig. 12) sinuous, sclerotized, apex with thin blunt point, vesica with two patches of thin setae.

Adult female (Fig. 2): similar to male. Forewing length 10 mm, no variation, n = 7. Genitalia (Figs 13, 14): Papilla analis not sclerotized, setose; posterior apophysis extends anteriorly to mid-point of eighth segment; anterior apophysis short and stout; eighth segment anterior ventral margin with two posteriorly directed processes, broadly rounded, acute apices; ductus bursae sclerotized at extreme posterior end, else membranous, elongate directed to right; corpus bursae expanded to left, signa absent.

#### Remarks.

 This new species is placed in the genus *Aleptina* Dyar (1902) based on appearance of the adult and the structure of the male and female genitalia.

#### Distribution and biology.

*Aleptina arenaria* occurs in White Sands National Monument, Otero County, New Mexico (Figs 17, 18). Adults were collected in black light traps placed within the white gypsum dunes, and interdunal areas. The immature stages and larval host plants are unknown.

## Discussion

In 2006 the U.S. National Park Service invited Metzler to initiate a long-term study of the Lepidoptera at White Sands National Monument, Otero County, New Mexico. A primary purpose of the study was to compile an inventory of moths in habitats within and immediately adjacent to the white gypsum dunes in the Monument.

White Sands National Monument preserves 285 km^2^ (110 mi^2^), or about 40%, of the world’s largest snow-white gypsum dune field. The remainder of the 275 square miles dune field is under the jurisdiction of the U.S. Army in the White Sands Missile Range. The dune field is located in the northern Chihuahuan Desert in southern New Mexico’s Tularosa Basin (Schneider–Hector 1993). A complete description of the study site and some of its unique biological resources is in Metzler et al*.* (2009).

There is a dearth of research on the invertebrate fauna in the gypsum dune field in the Tularosa Basin of New Mexico. Details of previous research pertinent to insects is in Metzler et al. (2009).

In the period 9 February 2007 through 30 September 2010 Metzler and Forbes identified more than 430 species of Lepidoptera (unpublished data) from the Monument. Because of the unusual physical and biological qualities of the New Mexico white gypsum dunes, we were especially aware of the possibility of finding undescribed species of moths. This is the fifth (Metzler et al. 2009, Metzler et al. 2010a, Metzler et al. 2010b, Metzler and Forbes 2011) in a series of papers pertinent to a detailed study of the Lepidoptera at White Sands National Monument, and this is the third species of moth described as part of this study. The study of Lepidoptera at White Sands National Monument by Metzler and Forbes is projected to last approximately 10 years.

**Figure:**
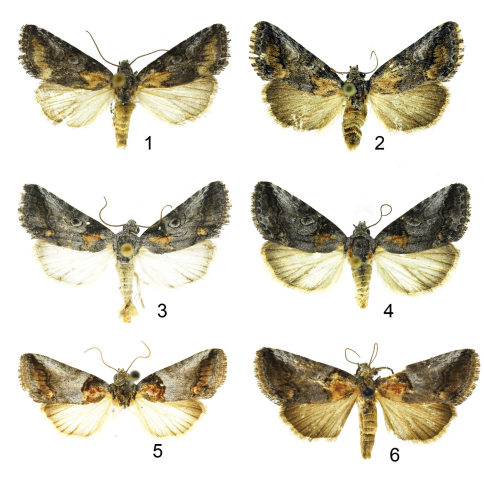
**Figures 1–6. *Aleptina* adults. 1** *Aleptina arenaria* Metzler & Forbes, male holotype **2** *Aleptina arenaria* Metzler & Forbes, female paratype **3** *Aleptina inca* Dyar, male **4** *Aleptina inca* Dyar, female **5** *Aleptina clinopetes* Dyar, male **6** *Aleptina clinopetes* Dyar, female.

**Figure:**
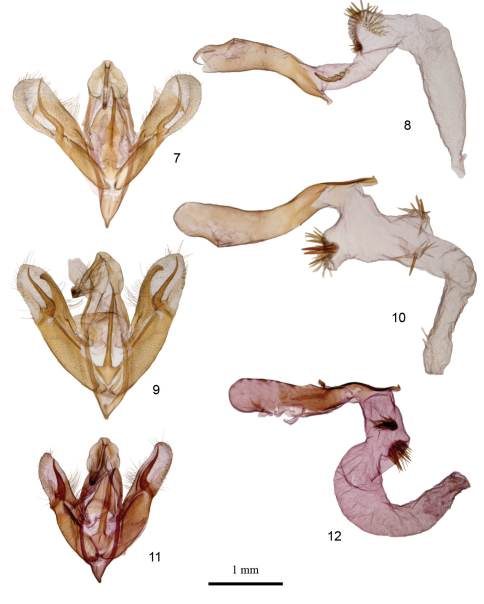
**Figures 7–12. *Aleptina* male genitalia. 7** *Aleptina inca* Dyar, male genital details **8** *Aleptina inca* Dyar, male genital details of aedeagus **9** *Aleptina clinopetes* Dyar, male genital details **10** *Aleptina clinopetes* Dyar, male genital details of aedeagus **11** *Aleptina arenaria* Metzler & Forbes, male genital details, paratype **12** *Aleptina arenaria* Metzler & Forbes, male genital details of aedeagus. paratype.

**Figure:**
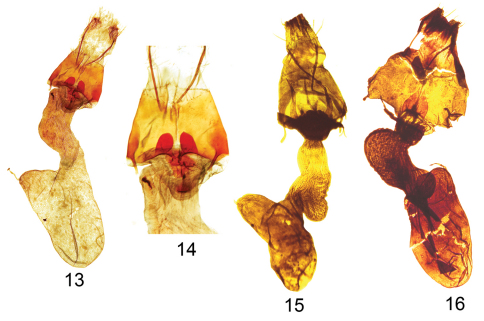
**Figures 13–16. *Aleptina* female genitalia. 13** *Aleptina arenaria* Metzler & Forbes, female genital details. paratype **14** *Aleptina arenaria* Metzler & Forbes, female genital details of two eighth sternite processes. paratype **15** *Aleptina inca* Dyar, female genital details **16** *Aleptina clinopetes* Dyar, female genital details.

**Figure:**
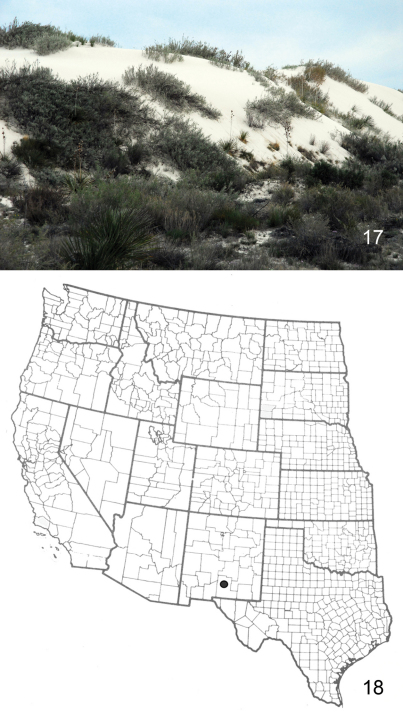
**Figures 17–18. *Aleptinaarenaria* habitat and distribution map. 17** White dunes habitat of type locality of *Aleptina arenaria* **18** Distribution map for *Aleptina arenaria*.

## Supplementary Material

XML Treatment for Aleptina arenaria
